# New Diagnostic and Therapeutic Approaches in Diabetic Microvascular Complications

**DOI:** 10.3390/biomedicines12081858

**Published:** 2024-08-15

**Authors:** Dragos Serban, Ana Maria Dascalu

**Affiliations:** 1 Faculty of Medicine, “Carol Davila” University of Medicine and Pharmacy, 020021 Bucharest, Romania; dragos.serban@umfcd.ro; 2 Fourth Surgery Department, Emergency University Hospital Bucharest, 050098 Bucharest, Romania; 3 Ophthalmology Department, Emergency University Hospital Bucharest, 050098 Bucharest, Romania

Diabetes mellitus is a major global health problem with an ascendant trend that makes it expected to reach up to 700 million cases by 2045 [1]. Population aging, obesity, and sedentarism, as well as the recent COVID-19 pandemic, are all risk factors that contribute to the increased incidence of this chronic, debilitating disease [2,3,4,5]. 

The microvascular triad unique to diabetes includes diabetic retinopathy, nephropathy, and neuropathy. Diabetic nephropathy is one of the most common causes of end-stage renal failure, and it is associated with having a severe impact on the patient’s quality of life [6,7]. Despite significant achievements in early diagnosis and therapy, diabetic retinopathy remains the leading cause of blindness in the working-age population [8]. Diabetic peripheral neuropathy affects nearly 50% of adults who have diabetes during their lifetime and represents a major risk factor for diabetic foot ulcer (DFU), the most common cause of non-traumatic amputations worldwide [9]. Classically, the duration of diabetes, the level of hyperglycemia, and the co-existence with arterial hypertension, as well as dyslipidemia, are related to the onset and progression of these invalidating diabetic complications. However, various training specialties and regular screening visits are required for the early diagnosis of these complications, placing a significant burden on healthcare systems worldwide. There is still a significant unmet need for novel diagnostic tools for early diagnosis, which is based on non-invasive, cost-effective screening methods that can provide personalized management for diabetic patients [10].

Novel research has found multiple molecular pathways that may interfere with vascular dysfunction, ischemia, and tissue damage. In this Special Issue, we showcase nine original articles and two comprehensive reviews that provide new data on novel biomarkers, early diagnosis, pathology, molecular mechanisms, and new therapies in the fields of diabetic retinopathy, nephropathy, neuropathy, and diabetic foot ulcers.

Postprandial plasma glucose (PPG), traditionally measured 1–2 h after a main meal, is a well-recognized clinical tool used not only in diabetes diagnosis but also in evaluating the risk of micro- and macrovascular diabetic complications, as well as therapeutic control of the disease [11]. In the first contribution to this Special Issue, Dr. Yutang Wang and col. examined a large number of patients included in the NHANES III study, which aimed to assess whether predicted PPG after 4–7.9 h could be used to diagnose diabetes. A multivariate prediction model considering 30 possible risk factors was developed to calculate predicted PPG after 4–7.9, and demonstrated a high diagnostic accuracy at 87.3%. The authors consider this parameter to be less influenced by diet or other confounders and suggest that PPG after 4–7.9 could be a promising diagnostic indicator for diabetes. 

Fedulovs et al., in the second article, and Hayden et al., in the third, provide new insights regarding the pathological changes in metabolic syndrome (MS) and diabetes. Meanwhile, in the study conducted by Fedulovs and col. (our ninth contribution), they performed for the first time a comparative analysis of the LPS, LBP, EndoCAb IgM, EndoCAb IgG, and fecal calprotectin in patients with T1D, as well as in controls, followed by a stratification of the results according to MS status. The authors evidence higher endotoxemia in T1DM patients with MS and reinforce the need for screening and treating MS in these patients. Dr. Melvin R Hayden and col. (contribution 3) provide a comprehensive review regarding the role of the perivascular unit, a novel concept developed by Troili and col. [12], in the development of neuroinflammation, cerebrovascular disease, and neurodegeneration encountered in metabolic syndrome, obesity, and T2DM. 

In the fourth paper, Dr. Ngema and Col. present in a narrative review, with evidence based on the Developmental Origins of Health and Disease (DOHaD) theory that T2DM during pregnancy could not only impact fetal development but also induce permanent impairment of hypothalamic–pituitary–adrenal (HPA) axis activity into adulthood. 

Skin autofluorescence (SAF) is a novel non-invasive biomarker on and invasive marker of advanced glycation end products [13]. The level of SAF describes the AGE accumulation in the body and is associated with its increased production, decreased degradation, and renal clearance. Several comparative studies have found that SAF values are increased in diabetic patients versus normal subjects and that skin autofluorescence is even higher when microvascular complications are present. However, there are still many challenges to be resolved, as SAF values appear to be influenced by skin pigmentation and the use of skin products; more studies on a larger number of patients are needed. In this context, we welcome the research of Dr. Reurean-Pintilei and Col. (contribution 5) on a large group of 885 T2DM patients. Their study found that SAF levels were correlated with HbA1c, diabetic kidney disease, and cardiovascular risk. A cut-off value of 2.36 was associated with very high CV risk, especially after adjusting for age, gender, and HbA1c level, and could be useful in selecting candidates for revascularization procedures.

Another approach to this challenging topic is that of Dr. Hiroki Yamagami and Col., whose study is featured in the sixth contribution to this Special Issue. A 1-year prospective study on 350 Japanese people with T2DM was carried out to comparatively evaluate the predictive power of SAF for the progression of diabetic kidney disease, by both urine albumin-to-creatinine ratio (uACR), as a biomarker of glomerular injury, and urine liver-type fatty acid-binding protein (uLFABP)-to-creatinine ratio (uL-FABPCR), as a biomarker of tubular injury. The authors found that SAF correlated with uL-FABP, but not with uACR, in people with T2D, suggesting that SAF can serve as a novel predictor for the development of diabetic tubular injury.

Angiopoietins and angiopoietin-like proteins are essential factors in angiogenesis, endothelium maturation, and inflammation [14,15]. Recently, these were investigated for their potential role in the endothelial dysfunction and inflammation processes encountered in diabetic nephropathy. Dr. Eman Alshawaf et al. (in contribution 7) quantified circulating ANGPTL3, ANGPTL4, ANGPTL8, Ang1, and Ang2 in the fasting plasma of patients with T2DM with and without diabetic nephropathy. They found a close association between Ang2 and ANGPTL8 in a population with DN, suggesting that they can function as DN risk predictors. In line with previous experimental and clinical evidence regarding the effects of Ang2 expression on podocytes through paracrine signaling, leading to glomerular EC destabilization and impaired filtration, this study highlights the importance of Ang2 as an early marker of tubular damage before the appearance of clinical symptoms like microalbuminuria.

A significant portion of this Special Issue is dedicated to novel insights into diabetic retinopathy. In the context of there being few specialists and an increased overload in ophthalmology departments due to screening visits, there is an increased need to develop simple screening tools that may be used by primary healthcare and diabetology departments to prioritize the patients who need urgent referrals to retina specialists. Serum inflammatory biomarkers based on a simple complete blood cell counts were investigated as potential predictive tools. There is solid evidence that neutrophil-to-lymphocyte ratio (NLR) and platelet-to-lymphocyte ratio (PLR) increase in T2DM, compared to normal subjects, and the values found in this regard are well correlated with the presence and the severity of diabetic complication [16,17,18]. These serum inflammatory biomarkers were thought to be correlated with chronic hyperglycemia and systemic inflammation levels. However, implementing those biomarkers in clinical practice is still challenging, due to the wide variation in means and cut-off values encountered in previous studies, and there is still a need for more evidence on the topic. The study conducted by Dr. Ana Dascalu et al. (this Special Issue’s eighth contribution) found that no statistical differences were noticed between NDR and NPDR groups for any of the white cell inflammatory biomarkers. However, significantly higher values for NLR, MLR, SII, and MPV were found in the PDR group when compared with the NDR and NPDR groups. This finding may signify that the level of systemic inflammation is higher in the advanced stage of DR associated with neovascularization. Age, sex, race, and ethnicity, as well as a large array of physiologic and pathological conditions, could impact serum inflammatory biomarkers. However, serum inflammatory biomarkers could be useful when they are integrated into comprehensive risk prediction models.

Optical coherence angiography (OCTA) is a novel imagistic approach that offers valuable information regarding macular vessels in a repeatable, non-invasive manner. Dr. Irini Chatziralli and col. (contribution 9) investigated changes in macular microvasculature using optical coherence tomography angiography (OCTA) in association with functional changes in patients with proliferative diabetic retinopathy (PDR) treated with pan-retinal photocoagulation (PRP) with a 12-month follow-up. The study results showed that at months 6 and 12 after PRP, foveal and parafoveal vessel density (VD) at the superficial capillary plexus (SCP) significantly increased compared to baseline, while the foveal avascular zone (FAZ) area significantly decreased and FAZ became more circular. The improvement of the choroidal circulation at the macula could be explained by the PRP-induced inflammatory response and the redistribution of choroidal flow from obliterated peripheral capillaries to the macula [19,20]. However, the authors could not find an improvement in BCVA in laser-treated patients.

Dr. Tanasie et al., in contribution 10, offer an interesting perspective on diabetic retinopathy as a neurodegenerative disease. In a comparative evaluation of the electrophysiological findings between patients with and without DR, the results indicate a substantial dysfunction of the neural retina in all stages of DR, with significant differences in response delays among study groups.

Intravitreal anti-VEGF is currently the mainstay therapy in diabetic retinopathy associated with macular edema. However, up to 30% of patients have a suboptimal response, suggesting that other molecules are involved in increased vascular permeability and capillary occlusion [21,22]. The valuable contribution of Dr. Lietuvninkas and Col. (contribution 11) is an experimental study that aims to analyze the “in vitro” properties of a novel agent: the (transforming growth factor beta) TGFβ receptor inhibitor RepSox (RS). The results of this study are encouraging, as RS performed better compared to anti-VEGF agents, not only in preventing barrier relaxation but also in promoting the closing of the relaxed barrier, induced by VEGF and TNF α. Thus, RS could be a more potent agent in treating diabetic macular edema by inhibiting multiple pathways.

Finally, we would like to take the opportunity to acknowledge all of these contributors for sharing their valuable work, taking a step forward in unveiling significant biological and molecular changes, and characterizing potential new tools for improving the diagnosis and management of diabetic microvascular complications.
